# Real-world treatment patterns and burden-of-disease of sub-optimally controlled hereditary angioedema^☆^

**DOI:** 10.1016/j.waojou.2025.101100

**Published:** 2025-09-01

**Authors:** Henriette Farkas, Emel Aygören-Pürsün, Didier G. Ebo, Noemi Bara, Fotis Psarros, Francois Gavini, Nawal Bent-Ennakhil, Laura Sayegh, Irmgard Andresen

**Affiliations:** a Semmelweis University, Budapest, Hungary; b University Hospital Frankfurt, Pediatric Clinic, Goethe University, Frankfurt, Germany; c Dept Immunology Allergology Rheumatology Antwerp University Hospital and Antwerp University, Belgium; d MediQuest Clinical Research Center, Sangiorgiu de Mures, Romania; e Navy Hospital of Athens, Athens, Greece; f EUCAN Medical Affairs, Takeda Pharmaceuticals International AG, Zürich, Switzerland; g Real-World Evidence, PPD, Part of Thermo Fisher Scientific, Montreal, Quebec, Canada

**Keywords:** Hereditary angioedema, Disease management, Burden of illness

## Abstract

**Background:**

Hereditary angioedema (HAE) is a rare disease characterized by unpredictable, recurrent subcutaneous or submucosal swelling that negatively impacts patients’ health-related quality of life (HRQoL). Despite treatment goals aimed at achieving complete control of the disease and normalizing patients' lives, the disease remains poorly controlled for some patients.

**Objectives:**

To describe the demographic, clinical and treatment characteristics, as well as the HRQoL impairment of patients with sub-optimally controlled HAE type I/II per treating physician's judgment, focusing on understanding the factors influencing the burden of illness.

**Methods:**

A chart review was conducted at 32 HAE care centers across 18 European countries, Canada, and Israel between April 2022 and January 2023 in 214 patients aged ≥12 years with HAE type I/II sub-optimally controlled with on-demand treatment (ODT) and/or long-term prophylaxis (LTP). Patients receiving lanadelumab were excluded, as it was not yet widely available during the eligibility period. A cross-sectional survey at patient enrollment included the Angioedema Quality of Life (AE-QoL) and EQ-5D-5L questionnaires to assess the impact of HAE on HRQoL.

**Results:**

Patients with uncontrolled HAE had a mean (standard deviation [SD]) of 9.9 (13) attacks per year, with a mean (SD) duration of 1.9 (1) days per attack. During the one-year observation period, 50.5% of patients were on ODT only, 36.0% used LTP and ODT concurrently, 6.5% used LTP without ODT, and 7.0% were untreated. Attenuated androgens (AA; stanozolol and danazol) in LTP were used by 24.7% of patients, while tranexamic acid (TA) and C1-esterase inhibitor (C1–INH) replacement products were used for LTP by 9.8% and 6.5%, respectively. The mean (SD) AE-QoL total score was 44.4 (24.0), indicating a moderate level of impairment, with women experiencing worse HRQoL (total score of 50.9 [SD 24] vs 37.3 [SD 23] in men) [where the minimal clinically important difference is 6 points] [1]. HRQoL worsened with increasing attack rates, from 41.4 (SD 24.0) among patients with one to 5 attacks/year, still indicating moderate impairment in HRQoL, to 73.0 (SD 27.0) for patients with >40 attacks/year.

**Conclusions:**

Suboptimal disease control in HAE was associated with the use of ODT only, as well as LTP mainly with AA/TA. It imposes a substantial burden on patients’ HRQoL, more particularly, but not exclusively, for those with frequent attacks and for women. The results suggest a need for improved HAE management.

ClinicalTrial.gov study identifier NCT04957641.

## Introduction

Hereditary angioedema (HAE) is a rare autosomal dominant disorder affecting 1 in 50,000 people worldwide. HAE types I and II are caused by missense mutations in the serpin family G member 1 gene (*SERPING1*). These mutations result in impaired synthesis (HAE type I) or impaired function (HAE type II) of the C1-esterase inhibitor (C1–INH) protein. HAE is characterized by the occurrence of painful, recurrent, and unpredictable attacks that primarily affect the subcutaneous and/or submucosal tissues of the extremities, face, abdomen, and larynx,^2^^,^^3^ usually requiring immediate medical intervention. The disease can result in significant physical, functional, and emotional burden for patients and their families.^4^

The ultimate treatment goal of HAE type I and II is to achieve total disease control and normalize patients’ lives. International guidelines recommend that patients be evaluated for long-term prophylaxis (LTP) at every visit, taking disease activity, burden, control, as well as patient preference into consideration; guidelines also suggest that all attacks be considered for on-demand treatment (ODT).^5^ Patients treated with ODT-only or LTP with attenuated androgens (AA)/tranexamic acid (TA) or C1–INH still suffer from inadequate or sub-optimally controlled disease; limitations include breakthrough attacks from limited effectiveness and substantial side effects for AA.

It is becoming increasingly important to consider the impact of HAE from the patient perspective in order to optimize treatment.^6^ In addition to the treatment's effectiveness and tolerability, health-related quality of life (HRQoL) has now become an important health outcome indicator and a primary focus of HAE guidelines.^7^

This study was conducted in 2022–2023 in Canada, Israel, and Europe. Its objective was two-fold: firstly, to determine the demographic, clinical, and treatment characteristics of patients with HAE type I and II who were experiencing poorly controlled HAE in real-world clinical practice; and secondly, to identify factors influencing disease burden, including HRQoL. At the time the study was launched, lanadelumab was not available in the market in most of the study countries. This gave us the opportunity to study the burden of uncontrolled disease when there are restrictions in access to recommended first-line care.

Validated tools were used to assess HRQoL components. The results from this study help to: (1) enhance awareness of treatment practices and disease burden associated with sub-optimally controlled HAE, and (2) underscore the importance of using HAE-specific HRQoL tools as physicians develop and evaluate treatment plans in HAE.

## Methods

A multicenter, observational medical chart-review study was conducted at 32 clinical centers specializing in HAE care across 18 countries in Europe (Austria, Belgium, Bulgaria, Croatia, Czech Republic, Germany, Greece, Hungary, Ireland, Latvia, Lithuania, Poland, Portugal, Romania, Serbia, Slovakia, Slovenia, and Spain), plus Canada and Israel.

### Eligibility criteria

Patients meeting the eligibility criteria at each clinical site were enrolled consecutively until the enrollment target was met. Patients with the most recent qualifying event date were given priority. Eligibility criteria included: (1) age 12 years or older, (2) physician-confirmed diagnosis of HAE type I or II, (3) occurrence of 1 or more HAE attack(s) within the eligibility period, (4) sub-optimally controlled disease according to the treating physician's judgment, (5) ability to comply with the study requirements, and (6) provision of informed consent or assent where required by local regulations. Patients enrolled in a trial of an investigational drug or device during the observation period or who had initiated LTP with lanadelumab at any time since diagnosis were excluded from the study.

### Study design and data collection

Staff at each clinical center abstracted anonymized data from the medical charts of patients with HAE type I or II related to their demographics, clinical characteristics, clinical HAE outcomes, and treatment using an electronic case report form. Ethics committee approvals and informed consent/assent were obtained in accordance with the local regulations. Patients could withdraw their consent/assent at any time for any reason.

The date of the most recent HAE attack relative to enrollment was considered the “qualifying event” date. The qualifying events for the patients fell between 07 October 2021 and 28 November 2022 (eligibility period). The observation period from which data were abstracted was defined as the 12-month period before the qualifying event date. The impact of HAE on HRQoL was measured at the time of enrollment (planned to be within 6 months of the qualifying event date) using validated self-report tools, including the Angioedema Quality of Life (AE-QoL) Questionnaire^8^ and EQ-5D-5L/EQ-5D-Y^9^ questionnaires. These questionnaires were made available to patients according to their age, language, and mode of administration approved in each specific country.

The AE-QoL addresses high-impact areas for the loss of HRQoL in patients aged ≥18 years with recurrent angioedema. It is composed of 17 questions regarding functioning, fatigue/mood, fear/shame (including appearance of swellings), and nutrition. Responses are based on a five-point Likert scale, ranging from “never” to “very often” with a recall period of 4 weeks. The raw scores were converted to percentages (ranging from 0 to 100), with higher scores indicating higher levels of impairment and lower HRQoL. Overall, the values define no effect (0–23), a small effect (24–38), or a moderate-to-large effect (≥39) on the HRQoL of patients with recurrent angioedema.^1^^,^^8^^,^^10^ The generic EQ-5D-5L descriptive system, composed of 5 dimensions (mobility, self-care, usual activities, pain/discomfort, and anxiety/depression), was assessed in patients aged ≥16 years. Each dimension was categorized into 5 levels: no problems, slight problems, moderate problems, severe problems, and extreme problems. Additionally, the visual analog scale (EQ-VAS) was employed to determine a global general health status score (100 indicating the best health status). The EQ-5D-5L norms reported for the EQ-VAS and for self-reported problems on each of the 5 dimensions of the EQ-5D-5L descriptive system, stratified by age and sex, were used as reference data for comparisons with HAE patients and the assessment of disease burden.^11^ The questionnaires were scored in accordance with the scoring algorithms of the respective instruments, with the necessary permissions from the copyright owners obtained for their use.

### Statistical analysis

Descriptive statistics were used to summarize patient demographic and clinical characteristics, therapeutic management of HAE (ie, ODT, LTP) and attack characteristics. The frequency of HAE attacks per month was calculated by dividing the number of attacks by the total observation period over which information on the frequency of HAE attacks was available (12 months).

Generalized linear models (GLMs) were used to investigate the association of HAE attack rate, HRQoL, and demographic/clinical characteristics. A Poisson distribution^12^ and a log link were used. Tweedie distributions were used for the model assessing risk factors for high attack rates.^13^ The distribution of the AE-QoL scores was initially inspected, and the Anderson-Darling test was performed to ascertain the normality of the data. The final choice for the distribution and link function was made based on goodness of fit and analysis of residuals. The following covariates were applied to the models: age (value), sex (male, female), disease duration (continuous), HAE type (I or II), and family history of C1–INH-HAE (yes/no). Furthermore, the presence of life-threatening attacks, severe attacks, and the administration of treatment were recorded. The type of treatment was classified as LTP and on-demand, on-demand only, or LTP only. The variables of interest, namely the use of C1–INH (yes/no), TA (yes/no), AA (yes/no), and attack rate, were incorporated into the model assessing risk factors for impaired HRQoL per AE-QoL total score. These variables were selected because of their potential clinical relevance.

Analyses were performed exclusively on observed data; no imputation was made for any missing values except for partial dates, where general imputation rules were applied. All analyses were performed using SAS software, version 9.4.2 (SAS Institute, Inc, Cary, North Carolina).

## Results

### Patient demographics and HAE attack characteristics

A total of 221 patients with HAE type I or II were enrolled in the study; however, 7 patients failed the screening process, leaving data from 214 patients included in the chart review population. Most patients (84.6%) were aged between 18 and 64 years (mean age 43.0 [standard deviation, SD, 16.0] years old; range 13–87 years old) at the time of their qualifying event. Most of the sample was female (58.9%), white (90.3%), had HAE type I (90.7%), and had a known family history of HAE (81.8%). The most commonly occurring comorbidities were hypertension (14.0% of patients), allergy/anaphylaxis (8.4%), anxiety (5.6%), asthma (5.1%), and depression (4.2%). The mean duration of HAE was 16.3 (SD 11.5) years (range: 0.2–49.5). A summary of demographic and clinical characteristics is shown in Table 1. Country-specific details are provided in Supplementary Table 1.

**Table 1:** Demographics and clinical characteristics of patients in BOISTERN

	Chart Review^a^ Population N = 214	HRQoL^b^ Population n = 138	AE-QoL Population n = 114	EQ-5D-5L Population n = 133
Age^c^ [years], mean (SD)	43.0 (16.0)	40.9 (16.0)	41.5 (15)	41.9 (16)
Range (min-max)	13–87	13–84	18–84	17–84
Female, n (%)	126 (58.9)	77 (55.8)	59 (51.8%)	75 (56.4%)
Type of HAE [I], n (%)	194 (90.7%)	126 (91.3%)	102 (89.5%)	121 (91.0%)
Disease duration^d^ [years], mean (SD)	16.34 (11.5)	16.72 (11.7)	17.63 (12.2)	16.94 (11.9)
Range	0.2, 49.5	0.2, 49.5	0.2, 49.5	0.2, 49.5
Family history of HAE, yes, n (%)	175 (81.8%)	116 (84.1%)	96 (84.2%)	111 (83.5%)
History of life-threatening HAE attacks, n (%)	21 (9.8%)	8 (5.8%)	7 (6.1%)	8 (6.0%)
Comorbid medical conditions >5%^e^, n (%)				
Hypertension	30 (14.0%)	15 (10.9%)	11 (9.6%)	15 (11.3%)
Allergy/anaphylaxis	18 (8.4%)	12 (8.7%)	10 (8.8%)	11 (8.3%)
Anxiety	12 (5.6%)	9 (6.5%)	9 (7.9%)	9 (6.8%)
Asthma	11 (5.1%)	5 (3.6%)	4 (3.5%)	5 (3.8%)
Depression	9 (4.2%)	7 (5.1%)	7 (6.1%)	7 (5.3%)
Heart disease	9 (4.2%)	8 (5.8%)	8 (7.0%)	8 (6.0%)
None	106 (49.5%)	67 (48.6%)	57 (50.0%)	64 (48.1%)

*AE-QoL,* Angioedema Quality of Life; *HAE*, hereditary angioedema; *HRQoL,* health-related quality of life; *SD*, standard deviation.

a Includes all patients with medical chart abstracted.

b Includes patients who completed the AE-QoL, EQ-5D-5L and/or EQ-5D-Y.

c At qualifying event.

d Derived by subtracting the date of diagnosis from the qualifying event date + 1 day.

e Conditions documented in the medical chart during the 12-month observation period. Categories are not mutually exclusive; therefore, the percentage can be greater than 100%.

**Table 2:** Mean AE-QoL scores, overall and across age and sex

AE-QoL Mean (SD)	Overall n = 114^a^	Age		Sex	
		18–64 n = 104	≥65 n = 10	Male n = 55	Female n = 59
Total score	44.4 (24)	44.0 (24)	47.8 (26)	37.3 (23)	50.9 (24)
Functioning score	43.7 (29)	43.5 (29)	45.8 (29)	37.6 (29)	49.5 (29)
Fatigue/mood score	38.5 (25)	38.1 (25)	42.5 (29)	33.0 (24)	43.6 (25)
Fears/shame score	53.4 (29)	53.2 (29)	55.9 (28)	44.6 (27)	61.7 (28)
Food score	33.4 (27)	32.7 (27)	41.5 (32)	26.8 (27)	39.6 (27)

*AE-QoL,* Angioedema Quality of Life; *SD,* standard deviation.

a Patients in the final AE-QoL analysis set.

**Fig. 1:**
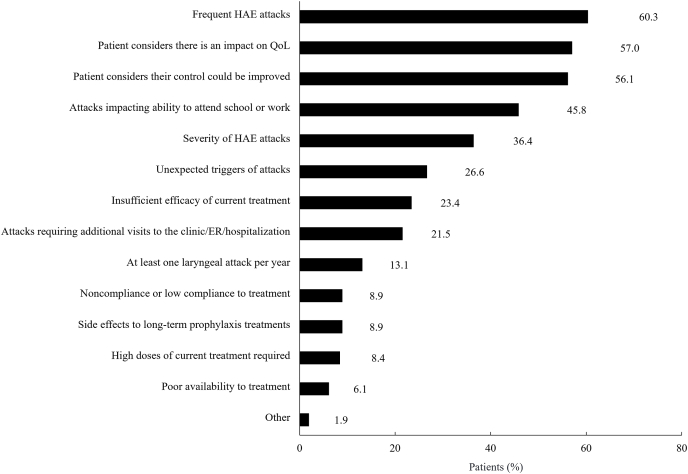
Reasons for sub-optimal/inadequate disease control of HAE disease control among patients. Treating HAE specialists assessed and provided reasons for their patients' inadequately controlled disease. Categories are not mutually exclusive; therefore, the percentage can be greater than 100. *HAE*, hereditary angioedema; *Pt*, patient; *QoL*, quality of life; *Tx*, treatment.

Physicians reported that frequent HAE attacks (60.3%), severity of HAE attacks (36.4%), impaired quality of life (57.0%), and inability to attend school or work (45.8%) were the primary reasons for insufficient disease control (inclusion criteria) (Fig. 1). For most patients (79.4%) suboptimal disease control was attributed to more than 1 factor, and 56.1% of patients believed that their disease control could be improved.

A total of 2127 HAE attacks were reported among the 214 patients over the course of the 12-month observation period. The mean number of attacks per patient was 9.9 (SD 13; range: 1–73) with a mean duration of 1.9 (SD 1) days per attack. The percentages of patients suffering from moderate attacks (activity limited mildly or moderately, some assistance may be needed) and severe attacks (activity considerably limited, assistance needed) were 76.6% and 45.3%, respectively. Also, 9.8% of patients experienced at least 1 life-threatening HAE attack, and 72.4% patients suffered, on average, from 5.7 (SD 11) attacks per year with prodromal symptoms. The prodromal symptoms most reported by patients included nausea (22.9%), erythema marginatum (15.9%), fatigue (15.4%), and malaise (12.1%). Patients, who could have reported attacks affecting different or multiple locations, experienced HAE attacks in their extremities (72.4%), followed by the abdomen (70.1%), the face (38.8%), and the larynx/tongue (25.7%). Most patients (81.3%) were able to identify the triggers of their HAE attacks. The most reported triggers were stress (61.2%), physical trauma (50.5%), infections (42.1%), exertion (35.5%), and food (10.3%).

A multivariate analysis revealed an association between HAE attack rate as well as sex and age. Women and younger patients (18–40 years) exhibited an increased risk of attacks compared to men and older (≥65 years) patients (*P* = 0.035 and *P* = 0.006, respectively) (Supplementary Table 2).

### Therapeutic management of HAE

During the 12-month observation period, ODT was used for 1439 of 2127 (67.7%) attacks by 185 (86.4%) patients. Of these patients, 108 (50.5%) used ODT without LTP treatment, while 77 (36.0%) used ODT and LTP simultaneously. A total of 14 (6.5%) patients did not use ODT concurrently with LTP. There was notable variation across countries regarding the proportion of patients using LTP at a given timepoint during the 12-month observation period, with rates ranging from 89.5% of patients in Portugal to 7.7% of patients in Lithuania. A total of 15 patients (7.0%) were not treated during the observation period. Of these, 7 were from Israel and 1 each was from Belgium, Hungary, Poland, Ireland, Spain, Lithuania, Greece, and Croatia.

The most frequently used ODTs among the 214 patients were bradykinin-receptor antagonists (58.4%; icatibant), followed by C1–INH replacement products (48.1%; intravenous [IV] pdC1-INH concentrate-Berinert [27.6%], IV pdC1-INH concentrate-Cinryze [7.9%], and rhC1-INH concentrate-Ruconest [15.4%]). AA (5.6%; danazol and stanozolol), TA (2.3%), and epsilon aminocaproic acid (0.9%) were taken less frequently.

On the other hand, AAs (danazol and stanozolol) were being used for LTP by 24.7% of patients, C1–INH replacement products by 7.5% of patients (IV pdC1-INH concentrate-Cinryze [6.1%]; subcutaneous pdC1-INH concentrate-Haegarda [0.5%], IV pdC1-INH concentrate-Berinert [0.9%]), and TA by 9.8% of patients. Desogestrel, epsilon aminocaproic acid, and berotralstat were used by 0.5%–2.8% of patients for LTP. The most common reasons for physicians to prescribe LTP were defined as “ODT was insufficient to control disease” (48.4%), “to improve quality of life” (29.7%), and “loss of adequate disease control to prior prophylactic treatment” (6.6%). Over the course of the 12-month study period, 84 (92.0%) patients did not alter their LTP regimen, with a mean duration on each medication of 8.1 (SD 9.62) years (Q1-Q3 1–9.79). LTP treatments were discontinued in 12 of the 91 (13.2%) patients, primarily due to poor tolerability (n = 4/12, 33.3%) and insufficient control of attacks (n = 3/12, 25.0%).

Although the attack burden was significant among patients on LTP, LTP use was associated with a reduction of attack rates (7.4 [SD 10] vs 11.8 [SD 14] HAE attacks per year among LTP vs non LTP users respectively), fewer severe attacks (40.7% vs 48.4% among LTP vs non LTP users respectively had severe attacks), and fewer attacks requiring ODT (4.9 [SD 7] vs 7.5 [SD 11] of LTP vs non LTP users, respectively, required ODT for attacks). Over the course of 12 months, 24.2% of patients on LTP had at least 1 emergency room (ER) visit related to an HAE attack, and 1.1% had at least 1 hospitalization related to an HAE attack. Similar estimates were observed among patients not taking LTP (19.5% and 3.3% HAE-attack related ER visits and hospital admissions, respectively).

### Patient-reported outcomes: HRQoL

The mean time elapsed between the qualifying event and completion of the HRQoL questionnaire was 76.5 (SD 64.0) days. Among the 114 (53.3%) patients included in the AE-QoL analysis population, the mean AE-QoL total score (with higher scores indicating higher levels of impairment and lower HRQoL), was 44.41 (SD 24.0), indicating a moderate level of impairment. Mean scores were highest for the fear/shame (53.4; SD 29.0), functioning (43.7; SD 29.0), and fatigue/mood (38.5; SD 25.0) domains (see Table 2).

**Fig. 2:**
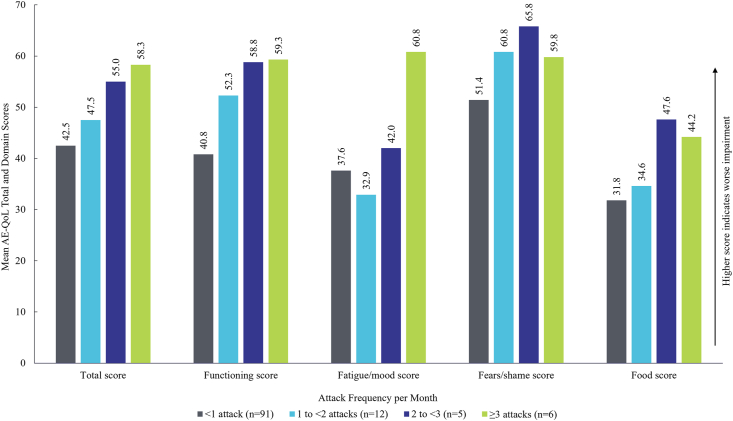
Mean AE-QoL total and domain score per attack frequency per month. *AE-QoL*, Angioedema Quality of Life.

Mean total scores worsened with increasing attack rates, ranging from 41.4 (SD 24.0) for patients with 1 to 5 attacks/year, still indicating moderate impairment in HRQoL, to 73.0 (SD 27.0) for those with >40 attacks/year. A similar trend was observed among domain scores (Fig. 2).

**Fig. 3:**
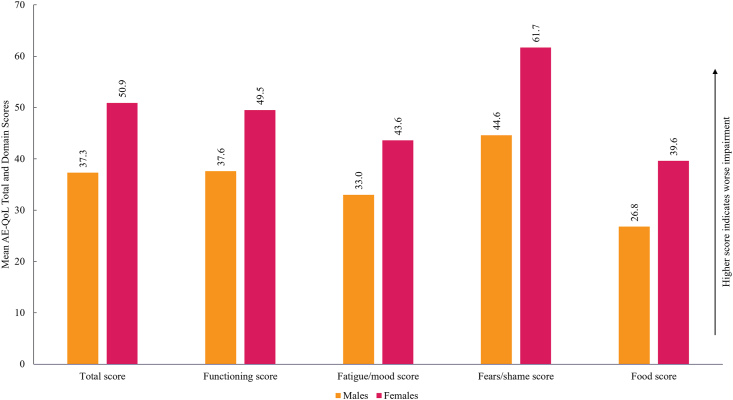
Mean Angioedema Quality of Life (AE-QoL) total and domain score by sex. *AE-QoL*, Angioedema Quality of Life.

Female participants reported greater impaired HRQoL in the AE-QoL total score compared to male participants (mean 50.9 [SD 24] vs 37.3 [SD 23], respectively) where the minimal clinically important difference is 6 points.^1^ This trend was also observed across each of the AE-QoL domains (Fig. 3). The highest AE-QoL domain score observed in women was for fear/shame. Multivariate analyses showed that being female was associated, on average, with an 11.19% increase in the total AE-QoL score compared to being male (95% confidence interval [CI] 2.23–20.15; *P* = 0.016) (Supplementary Table 3).

Most patients who were included in the EQ-5D-5L analysis population reported no problems with mobility (67.7%), self-care (85.0%), or usual activities (57.9%). Nevertheless, half of the patients reported problems with pain/discomfort (52.7%) and anxiety/depression (47.4%); reports of anxiety/depression tended to increase with an increasing number of attacks (data not shown). The EQ-VAS score was 73.9 (SD 18.0), indicating a slightly lower health status compared with the norm-based mean scores in this age group. Data for EQ-5D-Y are not reported due to the small sample size (n = 5 pediatric patients).

## Discussion

The present chart review of patients with sub-optimally controlled HAE describes real-world clinical practice across a large geographical area (Europe, Canada, and Israel) prior to the availability of lanadelumab.

The study demonstrates that uncontrolled HAE is a fluctuating and grave condition that may be life-threatening and negatively impact patients’ HRQoL. The patients were predominantly adults (aged ≥18 years) diagnosed with HAE type I, on average, 16 years before enrollment in the study. The patients reported approximately 10 HAE attacks per patient during the 12-month observation period, with most patients having experienced at least 1 documented moderate or severe HAE attack, with an average duration of 2 days. This indicates that disease activity remained high even after HAE was diagnosed and treated. About one-quarter of patients experienced at least 1 laryngeal HAE attack during the 12-month observation period. Acknowledging that our study population included those considered to be sub-optimally controlled, this is relatively high for such a timeframe, considering that the literature reveals that more than 50% of patients with HAE will experience at least 1 laryngeal attack during their lifetime.^6^ The majority (86.4%) of patients with uncontrolled HAE used ODT in case of an attack in accordance with international guidelines.^5^ However, during the observation period, more than half of the patients received ODT-only compared to 36.0% who received both LTP and ODT, and 6.5% who received LTP without ODT.

The use of AA as LTP was common, and TA was prescribed to 10% of patients, despite the well-documented substantial side effects of the former (eg, weight gain, muscle cramps, seborrhea, acne, and virilization in women) and the lack of data on the efficacy of the latter. Importantly, several HAE guidelines do not recommend either AA or TA as first-line therapy.^5^^,^^14^^,^^15^ Patients on LTP with AA/TA still experienced a high frequency of attacks and had a similar number of HAE attack-related ER visits and hospitalizations as those on OD only.

Our findings align with current guidelines on the importance of considering HRQoL outcomes in addition to clinical outcomes as physicians monitor LTP in HAE.^6^ Regardless of the number of attacks, patients still have impaired HRQoL, particularly associated with feelings of fear/shame. It is possible that the unpredictable character of attacks may pose a burden, even when HAE disease activity is low; patients may live in persistent anxiety and fear about attacks (especially a laryngeal attack), passing HAE to children, reduced work/school productivity, and limited career/educational achievement.^16^ This is supported by the fact that half of patients in this study self-reported problems with anxiety/depression, suggesting that patients are affected not only at physical level but also emotionally and psychologically. Overall, our findings suggest that relying solely on attack frequency and severity could significantly underestimate the impact of HAE on the lives of patients.^6^

Our study also aligns with published literature supporting a higher frequency of type I and II HAE among women. As in other studies,^17^, ^18^, ^19^, ^20^, ^21^ this study suggests that women with HAE may require more catered treatment because fluctuations in female sex hormones (eg, changes that occur during puberty, menses, contraceptive use, pregnancy, and menopause) can affect the frequency and severity of attacks.^22^, ^23^, ^24^ In addition to poorer clinical disease control, women reported 11% greater HRQoL impairment than men on the AE-QoL tool, which could be related to HAE potentially affecting aspects of gynecologic care and vice versa.

This study found that the impact of HAE on HRQoL was lower using the generic EQ-5D-5L tool compared to the disease-specific AE-QoL tool. Despite the ability of generic questionnaires to compare HRQoL across different diseases, it is well known that they generally have low sensitivity when assessing specific aspects of the disease, which limits their clinical utility. Therefore, our study supports the use of disease-specific tools instead of generic instruments to assess HRQoL in HAE clinical practice.^25^

Strengths of this study include its geographical representativeness of several countries, as well as confirmation of data through patient medical charts from real-life practices and HRQoL surveys. So, unlike some studies evaluating the humanistic impact of HAE,^26^, ^27^, ^28^, ^29^ this study uniquely combines patient-reported outcome measures with a traditional medical record review, providing a comprehensive perspective on the burden of HAE. Finally, despite HAE being a rare disease, the study had a relatively large sample size.

Limitations include the incompleteness of individual attack records at the participating clinical centers, which may have underestimated the attack rate in HAE, and the relatively small country-specific sample sizes, which precluded the evaluation of geographical differences (eg, with respect to the availability and accessibility of LTP therapies). There is a risk that unmeasured confounding variables may have affected the analysis, despite the adjustments that were made using multivariable analyses. Lastly, the exclusion of lanadelumab users, due to its unavailability or limited availability during the eligibility period, prevented the analysis of the clinical burden associated with lanadelumab treatment. A separate study addresses this gap in knowledge.^30^

## Conclusions

Overall, findings from this study suggest that patients with uncontrolled HAE who were treated prior to December 2022 predominantly used ODT-only or LTP, mainly with AA/TA treatments, and were experiencing substantial disease burden, as measured by the validated HAE-specific HRQoL tool. Our data, reflecting real-life treatment practices prior to the availability of lanadelumab, show that therapies like danazol, stanozolol, and TA were commonly used, despite other available LTP treatments (eg, Cinryze, Berinert subcutaneous 2000/3000). This finding underscores the necessity to adhere to international guidelines, promoting/recommending utilization of more efficacious and well-tolerated therapies. The study also found that some patients with low attack rates have markedly impaired quality of life, which aligns with current guideline recommendations to monitor HRQoL to optimize LTP treatment.

## Availability of data and materials

The data that support the findings of this study may be available upon request from the corresponding author.

## Author contributions

**Henriette Farkas:** Investigation, Writing – Original Draft, Writing - Review & Editing; **Emel Aygören-Pürsün**: Investigation, Writing – Original Draft, Writing - Review & Editing; **Didier G Ebo**: Investigation, Writing – Original Draft, Writing - Review & Editing; **Noemi Bara**: Investigation, Writing – Original Draft, Writing - Review & Editing; **Fotis Psarros**: Investigation, Writing – Original Draft, Writing - Review & Editing; **Francois Gavini**: Conceptualization, Methodology, Writing – Original Draft, Writing - Review & Editing; **Nawal Bent-Ennakhil**: Conceptualization, Methodology, Validation, Writing – Original Draft, Writing - Review & Editing; **Laura Sayegh**: Methodology, Validation, Writing – Original Draft, Writing - Review & Editing; **Irmgard Andresen**: Conceptualization, Methodology, Validation, Writing – Original Draft, Writing - Review & Editing, Supervision, Funding Acquisition.

## Ethics, consent and permissions statement

Ethics committee approvals were obtained in all countries according to local regulations. Patients who were eligible and provided consent/assent were enrolled in the study. ClinicalTrial.gov study identifier NCT04957641.

## Submission declaration

The research described in this article has not been published previously, it is not under consideration for publication elsewhere, and its publication is approved by all authors and tacitly or explicitly by the responsible authorities where the work was carried out. If accepted, this work will not be published elsewhere in the same form, in English or in any other language, including electronically without the written consent of the copyright holder.

## Funding

This study was funded by 10.13039/100016469Takeda Pharmaceuticals International AG. PPD worked on data collection, analysis, interpretation of data and report writing. Takeda Pharmaceuticals International AG conceptualized the study design and oversaw PPD's study conduct. Medical writing support, under the direction of the authors, was performed by Maria Angeles Natividad Sancho, PhD (PPD), funded by 10.13039/100016469Takeda Pharmaceuticals International AG.

## Author's consent for publication

The final submitted manuscript has been seen and approved by all the authors.

## Declaration of competing interest

Henriette Farkas reports receiving research grants from CSL Behring, Shire/Takeda and Pharming and consultancy/speaker fees and honoraria from BioCryst Pharmaceuticals, Inc., CSL Behring, Pharming Group NV, KalVista and Shire HGT/Takeda and serves as an advisor and principal investigator for clinical trials/registries for BioCryst Pharmaceuticals, Inc., CSL Behring, Pharming, KalVista and Shire/Takeda.

Emel Aygören-Pürsün received grants and/or fees as consultant or speaker for Biocryst, Centogene, CSL Behring, Kalvista, Pharming, Pharvaris and Shire/Takeda.

Didier G Ebo received grants/fundings and/or fees as consultant or speaker for Biocryst, CSL Behring, Ionis Pharmaceuticals, KalVista, Takeda.

Noemi Bara has received research grants from Takeda and Pharming and consultancy/speaker fees and honoraria from Pharming Group NV, KalVista and Shire HGT/Takeda and serves as an advisor and principal investigator for clinical trials for BioCryst Pharmaceuticals, Pharming, KalVista and Shire/Takeda.

Fotis Psarros has received speaker and advisory board funding from Takeda Pharmaceuticals, CSL Behring and principal investigator for clinical trials/registries for CSL Behring, Pharming, KalVista and Shire/Takeda.

Francois Gavini, Nawal Bent-Ennakhil and Irmgard Andresen are employees of Takeda and own Takeda stocks.

Laura Sayegh is an employee of PPD, part of Thermo Fisher Scientific, Canada.
