# Supplementary material for: Real-world treatment patterns and burden-of-disease of sub-optimally controlled hereditary angioedema
Source: World Allergy Organ J. 2025 Sep 1;18(9):101100. doi: 10.1016/j.waojou.2025.101100 (PMC12423343; doi:10.1016/j.waojou.2025.101100)
Supplement: Multimedia component 1 [file mmc1.docx]

**Supplementary Table 1. Demographics and clinical characteristics of patients in BOISTERN, by Country**

| **Country, n patients** | **Age^a^ (years) Mean (SD) (range)** | **Gender (Female), n (%)** | **HAE Type I, n (%)** | **Disease duration^b^ (years)  Mean (SD) (range)** | **Family**  **History of HAE, n (%)** |
| --- | --- | --- | --- | --- | --- |
| Austria, n=7 | 51.0 (14) (37, 76) | 3 (42.9%) | 7 (100.0%) | 18.40 (11.8) (1.2, 33.)6 | 4 (57.1%) |
| Belgium, n=11 | 46.4 (15) (30, 67) | 5 (45.5%) | 8 (72.7%) | 12.40 (9.5) (0.2, 32.7) | 10 (90.9%) |
| Bulgaria, n=10 | 48.8 (13) (27, 77) | 5 (50.0%) | 10 (100.0%) | 10.13 (11.3) (2.9, 41.9) | 10 (100.0%) |
| Canada, n=5 | 42.8 (8) (30, 50) | 3 (60.0%) | 5 (100.0%) | 20.80 (11.0) (9.0, 35.3) | 5 (100.0%) |
| Croatia, n=15 | 42.8 (17) (19, 74) | 11 (73.3%) | 13 (86.7%) | 19.39 (14.9) (0.9, 37.7) | 12 (80.0%) |
| Czech Republic, n=9 | 48.6 (19) (21, 76) | 6 (66.7%) | 8 (88.9%) | 21.21 (14.3) (1.2, 41.8) | 9 (100.0%) |
| Germany, n=2 | 28.5 (18) (16, 41) | 2 (100.0%) | 1 (50.0%) | 11.85 (15.6) (0.8, 22.9) | 1 (50.0%) |
| Greece, n=19 | 40.8 (11) (14, 58) | 6 (31.6%) | 18 (94.7%) | 19.35 (7.9) (8.6, 34.9) | 17 (89.5%) |
| Hungary, n=15 | 39.5 (17) (13, 70) | 7 (46.7%) | 15 (100.0%) | 18.99 (10.6) (0.7, 40.0) | 11 (73.3%) |
| Ireland, n=9 | 33.7 (12) (18, 50) | 4 (44.4%) | 5 (55.6%) | 22.51 (17.2) (3.8, 43.7) | 9 (100.0%) |
| Israel, n=20 | 38.3 (22) (14, 84) | 8 (40.0%) | 19 (95.0%) | 21.46 (13.4) (1.9, 49.5) | 18 (90.0%) |
| Latvia, n=6 | 55.0 (11) (35, 66) | 6 (100.0%) | 6 (100.0%) | 9.92 (6.8) (1.0, 16.7) | 4 (66.7%) |
| Lithuania, n=13 | 49.8 (18) (20, 81) | 10 (76.9%) | 13 (100.0%) | 11.53 (8.0) (2.8, 26.8) | 8 (61.5%) |
| Poland, n=7 | 40.6 (10) (30, 59) | 7 (100.0%) | 7 (100.0%) | 6.73 (5.7) (1.3, 18.4) | 6 (85.7%) |
| Portugal, n=19 | 46.0 (15) (18, 68) | 12 (63.2%) | 17 (89.5%) | 12.49 (8.9) (0.5, 32.6) | 18 (94.7%) |
| Romania, n=11 | 36.4 (10) (18, 53) | 9 (81.8%) | 9 (81.8%) | 8.62 (5.7) (0.8, 16.2) | 6 (54.5%) |
| Serbia, n=10 | 45.4 (16) (22, 76) | 9 (90.0%) | 10 (100.0%) | 18.42 (12.0) (4.6, 42.5) | 8 (80.0%) |
| Slovakia, n=7 | 32.0 (12) (15, 49) | 2 (28.6%) | 5 (71.4%) | 12.00 (3.0) (8.3, 16.4) | 6 (85.7%) |
| Slovenia, n=4 | 51.8 (15) (40, 73) | 2 (50.0%) | 4 (100.0%) | 18.93 (15.0) (0.7, 36.8) | 3 (75.0%) |
| Spain, n=15 | 42.7 (22) (16, 87) | 9 (60.0%) | 14 (93.3%) | 19.43 (10.2) (0.7, 32.2) | 10 (66.7%) |

^a^ At qualifying event

^b^ Derived by subtracting the date of diagnosis from the qualifying event date + 1 day

**Supplementary Table 2. Association of baseline factors with number of HAE attacks**

|  | **Patients n=214** | **Percent Change**  **95% CI Lower** | **Percent Change**  **95% CI Higher** | **Rate Ratio** | **Rate Ratio 95% CI Lower** | **Rate Ratio 95% CI Higher** | **P-value** |
| --- | --- | --- | --- | --- | --- | --- | --- |
| Sex (male) | 88 (41.1%) | -56.5% | -3.0% | 0.65 | 0.435 | 0.97 | 0.0347 |
| Sex (female) (ref.) | 126 (58.9%) |  |  |  |  |  |  |
| Age (40 to <65 years) | 97 (45.3%) | -23.1% | 90.2% | 1.209 | 0.769 | 1.902 | 0.4105 |
| Age (<18 years) | 9 (4.2%) | -69.9% | 87.3% | 0.751 | 0.301 | 1.873 | 0.539 |
| Age (≥65 years) | 24 (11.2%) | -74.4% | -20.2% | 0.452 | 0.256 | 0.798 | 0.0062 |
| Age (18 to <40 years) (ref.) | 84 (39.3%) |  |  |  |  |  |  |
| Disease duration (years) | 213 (99.5%) | -3.5% | 0.3% | 0.984 | 0.965 | 1.003 | 0.1053 |
| HAE type (II) | 20 (9.3%) | -69.7% | 4.6% | 0.563 | 0.303 | 1.046 | 0.0689 |
| HAE type (I) (ref.) | 194 (90.7%) |  |  |  |  |  |  |
| Family history of C1-INH-HAE (yes) | 175 (81.8%) | -71.0% | 1.3% | 0.542 | 0.29 | 1.013 | 0.0547 |
| Family history of C1-INH-HAE (no/unknown) (ref.) | 39 (18.2%) |  |  |  |  |  |  |

*C1-INH,* C1-esterase inhibitor; *CI,* confidence interval; *HAE*, hereditary angioedema; *LTP,* long-term prophylaxis

The table shows the effect of baseline factors on the rate of HAE attacks during the observation period.

Akaike information criterion =1359.0749

N events=2127

Tweedie distribution with log link function

**Supplementary Table 3. Association of baseline factors with AE-QoL total score**

|  | **Patients n=114** | **Mean Change** | **Mean Change**  **95% CI Lower** | **Mean Change**  **95% CI Higher** | **P-value** |
| --- | --- | --- | --- | --- | --- |
| Sex (female) | 59 (51.8%) | ref. |  |  |  |
| Sex (male) | 55 (48.2%) | -11.19 | -20.147 | -2.234 | 0.0161 |
| Age (18 to <40 years) | 54 (47.4%) | ref. |  |  |  |
| Age (40 to <65 years) | 50 (43.9%) | 6.884 | -2.898 | 16.665 | 0.171 |
| Age (≥65 years) | 10 (8.8%) | 5.357 | -12.875 | 23.588 | 0.566 |
| Disease duration (years) | 113 (99.1%) | -0.012 | -0.456 | 0.432 | 0.9576 |
| HAE type (I) | 102 (89.5%) | ref. |  |  |  |
| HAE type (II) | 12 (10.5%) | -1.697 | -16.141 | 12.747 | 0.8184 |
| Family history of C1-INH-HAE (no/unknown) | 18 (15.8%) | ref. |  |  |  |
| Family history of C1-INH-HAE (yes) | 96 (84.2%) | -9.759 | -22.745 | 3.227 | 0.144 |
| Life-threatening attack(s) during the observation period (no) | 107 (93.9%) | ref. |  |  |  |
| Life-threatening attack(s) during the observation period (yes) | 7 (6.1%) | 10.525 | -8.35 | 29.399 | 0.2771 |
| Severe attack(s) during the observation period (no) | 66 (57.9%) | ref. |  |  |  |
| Severe attack(s) during the observation period (yes) | 48 (42.1%) | 3.088 | -7.119 | 13.294 | 0.5546 |
| Neither LTP nor on-demand | 9 (7.9%) | ref. |  |  |  |
| On-demand only | 57 (50.0%) | 7.837 | -9.677 | 25.351 | 0.3826 |
| On-demand with LTP | 40 (35.1%) | -27.278 | -56.558 | 2.002 | 0.0709 |
| LTP only | 8 (7.0%) | -14.347 | -41.628 | 12.934 | 0.3052 |
| C1-INH (no) | 107 (93.9%) | ref. |  |  |  |
| C1-INH (yes) | 7 (6.1%) | 50.37 | 19.524 | 81.217 | 0.0019 |
| Tranexamic acid (no) | 101 (88.6%) | ref. |  |  |  |
| Tranexamic acid (yes) | 13 (11.4%) | 29.576 | 3.289 | 55.864 | 0.0298 |
| Androgens (no) | 87 (76.3%) |  |  |  |  |
| Androgens (yes) | 27 (23.7%) | 30.147 | 7.136 | 53.158 | 0.0118 |
| Number of HAE attacks during the observation period | 114 (100.0%) | 0.059 | -0.338 | 0.456 | 0.7701 |

*C1-INH,* C1-esterase inhibitor; *CI,* confidence interval; *HAE*, hereditary angioedema; *LTP,* long-term prophylaxis

Akaike information criterion=718.63500954

Normal distribution with identity link function
